# A genetic approach to comprehend the complex and dynamic event of floral development: a review

**DOI:** 10.5808/gi.21075

**Published:** 2022-12-30

**Authors:** Jatindra Nath Mohanty, Swayamprabha Sahoo, Puspanjali Mishra

**Affiliations:** 1 Department of Botany, School of Applied Sciences, Centurion University of Technology and Management, Bhubaneswar, 752050, India; 2 Centre for Biotechnology, SOA Deemed to be University, Bhubaneswar, Odisha 751003, India; 3 Department of Dietetics & Nutrition, IMS and Sum Hospital, SOA Deemed to be University, Bhubaneswar, Odisha 751003, India

**Keywords:** ABCDE model, floral genetics, next-generation sequencing, sex-linked gene, transcription factor

## Abstract

The concepts of phylogeny and floral genetics play a crucial role in understanding the origin and diversification of flowers in angiosperms. Angiosperms evolved a great diversity of ways to display their flowers for reproductive success with variations in floral color, size, shape, scent, arrangements, and flowering time. The various innovations in floral forms and the aggregation of flowers into different kinds of inflorescences have driven new ecological adaptations, speciation, and angiosperm diversification. Evolutionary developmental biology seeks to uncover the developmental and genetic basis underlying morphological diversification. Advances in the developmental genetics of floral display have provided a foundation for insights into the genetic basis of floral and inflorescence evolution. A number of regulatory genes controlling floral and inflorescence development have been identified in model plants such as *Arabidopsis thaliana* and *Antirrhinum majus* using forward genetics, and conserved functions of many of these genes across diverse non-model species have been revealed by reverse genetics. Transcription factors are vital elements in systems that play crucial roles in linked gene expression in the evolution and development of flowers. Therefore, we review the sex-linked genes, mostly transcription factors, associated with the complex and dynamic event of floral development and briefly discuss the sex-linked genes that have been characterized through next-generation sequencing.

## Introduction

Understanding the dynamics of sexual manifestations has enormous significance both in classical and theoretical research. Hermaphroditism is the major sexual system in the plant kingdom, with male and female sexual structures coexisting in the same flower. The XY and WZ sex determination systems commonly found in animals have hardly developed in plants, and are recognized in only a few genera of flowering plants [1]. Additionally, dioecy or isolated sexes have developed in around 7% of all angiosperms [2], typically from complete-flowered or monoecious descendants [3]. Variation in the outcomes of sexual reproduction of plants establishes outcrosses, which are essential for promoting inherent disparities and improving the adaptability of plant types. It remains unclear whether all dioecious organisms have a characteristic sexual morphology. However, theoretical findings indicate that sex-linked genes specifically accumulated in the recombination-suppressed regions of one of the gonosomes. This accumulation of gonosome-specific genes resulted in the corporeal differentiation of the masculine and feminine sex chromosomes [4]. More precisely, the evolution of sexual patterns was initiated with the expression of sex-linked genes within a recombination-suppressed region of a chromosome.

The concepts of phylogeny and floral genetics play a crucial role in understanding the origin and diversification of flowers in angiosperms [5]. Out of 25,500 (Approx) genes in Arabidopsis, more than 20,000 genes are responsible for pollen development at some point in time [6]. Research on the genetics and molecular biology of floral development and sex differentiation has resulted in the discovery of various floral identity genes. The majority of these genes belong to a smaller set of regulators called transcription factors (TFs), which govern the complexity of the transition from the floral meristem to the mature flower, as well as deciphering of the sexes. Floral organ speciation (sepals, petals, stamens, and carpels) and sexual variability are combined activities of floral homeotic genes, according to the ABC model [7]. Our aim here is to present a detailed review of sex-linked genes, including TFs and others that have been found to be involved in sex differentiation or floral development.

## Sex-Linked Genes in Flowering Plants

Phenotyping of floral mutants and their associated genetic interaction studies have shown that A function genes alone specify sepals, while the combined activity of A and B class genes leads to petals. Stamen development reflects the combined contributions of the B and C genes, while C functioning alone leads to carpels [7,8]. A comprehensive re-evaluation of the ABC model then led to the inclusion of the E function genes (the ABCE model), which are associated with the speciation of all organ types [9,10]. Subsequently, the D class genes were added, which determine the feature of ovule development in female flowers. These floral-specific genes code for the MADS-box family of TFs and are highly diversified in plants [11]. Based on their characterization in *A. thaliana*, *APETALA1 (AP1)* and *APETALA2 (AP2)* are A function genes, the B function is contributed by *APETALA3 (*AP3*)* and *PISTILLATA (PI)*, the C function is encoded by *AGAMOUS (AG)*, and the E function is carried out by multiple *SEPALLATA (SEP)* genes (i.e., *SEP1* to *SEP4*) [12].

In *Silene latifolia*, the Y chromosome-linked *Sl*AP3** gene encodes the *Apetala 3* MADS box protein in male flowering buds [13]. *Sl*AP3** exhibited high similarity with the *Arabidopsis* floral identity gene *AP3*, which is responsible for floral morphogenesis and organ identity [14]. Another Y-linked sequence named CCLS96 from *S. latifolia* encoding multiple copy numbers of non-coding RNAs has been found to be responsible for male bud expression [15]. X-linked genes in *S. latifolia*, such as *MROS3X* and *SlMF1*, demonstrated significantly high expression in female floral buds [13,16]. A few XY-linked genes, such as SlssX/SlssY, SlX1/SlY1, SlX3/SlY3, SlX4/SlY4, and DD44X/Y, have also been characterized in *S. latifolia* and are believed to have housekeeping functions [17]. A list of the sex-linked genes identified in different plant species is presented in Table 1.

## TFs and the Regulation of Flower Development

TFs are a group of regulatory proteins that play critical roles in altering the expression of genes associated with cellular pathways and biological processes, including sex differentiation, floral development, and the floral transition [18]. A wide range of TFs are known as major determinants of sex speciation in angiosperms. The most prominent are the MADS-box family TFs, which play important roles in many aspects of plant growth and are crucially involved in floral organ speciation and reproductive development [19]. These proteins are characterized by the presence of a 58–60 amino acid-long conserved MADS-box DNA binding domain at the N-terminus that dimerizes to specific DNA sequences called “CArG boxes” [20]. Based on protein domain structures, the MADS-box genes have been divided into two lineages: type I and type II. The type I or M-type gene forms a heterogeneous group with short DNA sequences (≈ 180 bp) encoding only the MADS domain and are classified as Mα, Mβ, and Mγ based on phylogeny. Although they constitute the major component of MADS-box genes in many plants, their functional attributes have only been characterized recently [21]. The type II or MIKC genes are characterized by the presence of additional domains, including an intervening (I) domain, a keratin-like (K) domain, and a C-terminal (C) domain. They are classified as canonical (MIKCC) or star type (MIKC*) depending on the alteration of their motif structure. Additionally, MIKCC genes are further divided into 14 clades based on phylogenetic relationships and distinct sequence motifs in their C-terminal domains [22]. An alteration in the C-terminal motif results in the transcriptional activation of specific DNA sequences through the formation of multimeric MADS-box protein complexes [19]. The MIKC subfamilies are often conserved and exhibit similar functions in the growth of different plants, as well as in reproductive and vegetative speciation, such as in the differentiation of the floral meristem (*APETALA 1* [*AP1*], *FRUITFUL* [*FUL*], and *CAULIFLOWER*), the development of floral organs (*AP1, APETALA3* [*AP3*], *PISTILLATA* [*PI*], *AGAMOUS* [*AG*], and *SEPALLATA 1-3* [*SEP1-3*]), the regulation of flowering time (*SUPPRESSOR OF OVEREXPRESSION OF CONSTANT 1* [*SOC1*], *FLOWERING LOCUS C* [*FLC*], *SHORT VEGETATIVE PHAGE* [*SVP*], *AGAMOUS-LIKE 24* [*AGL24*]), fruit maturation (*SHATTERPROOF 1-2* [*SHP1-2*]), embryonic development (*TRANSPARENT TESTA 16* [*TT16*]), and root growth (*AGAMOUS-LIKE 17* [*AGL17*]) (reviewed in Smaczniak et al. 2012; Theiben et al. 2016) [19,20]. The key function of MIKCC genes is classified into five classes: A, B, C, D, and E, each with multiple MADS-box TFs directly involved in the development of the floral quartet model [19,20]. The ABCDE model accounts for the regulation of flowering plants. Several reports have shown that MIKCC genes are fundamental to organogenesis, such that the combination of A + E genes identifies sepals, A + B + E specifies petals, B + C + E yields stamens, C + E gives carpels, and C + D + E specifies ovules. In contrast, MIKC* genes have been implicated in floral transition and gametophytic development [19]. Moreover, the *FLC* subfamily of genes also plays a role in controlling flowering through vernalization in *A. thaliana* [23]. *AGL12* has been implicated in pigment accumulation and root development in the floral transition in rice and proliferation of the root meristem in *Arabidopsis* [24]. Similarly, *TM8* genes control flower development in tomatoes and grapevines [25]. Most of the SOC family proteins act as activators, whereas *SVP*-related genes act repressors of floral patterning and the floral meristem in both monocots and dicots [26,27].

In addition to the MADS-box family of proteins, TFs encoding zinc fingers also function as major components of the architecture for plant development and organ differentiation [28]. The expression of *CmWIP1*, a sex-linked gene encoding C_2_H_2_ zinc finger protein, has been found to cause carpel abortion and the development of male flowers in *Cucumis melo* [29]. The MYB family of TFs plays a significant role in plant development and sex differentiation, as has been established in *A. thaliana*. AtMYB21, AtMYB24, AtMYB57, AtMYB108/BOS1, AtMYB35/TDF1, AtMYB80, and AtMYB99 are independently and individually responsible for anther development and/or function [30,31]. AtMYB33 and AtMYB65 assist in both anther and pollen development [32]. AtMYB115 and AtMYB118 are associated with embryogenesis [33], while AtMYB125 controls male germ cell division and differentiation [34]. AtMYB105 and AtMYB117 control lateral organ separation and axillary meristem formation [35]. Recently, an MYB-like gene (male-specific expression [MSE1]) linked to early anther development has been isolated from *Asparagus officinalis* [36]. Homeodomain-leucine zippers (HD-Zips) are a specific group of plant TFs with significant role in plant development, floral differentiation, and embryogenesis [37]. MeGI, an HD-Zip gene, acts as a regulatory factor for anther fertility and as a major sex determinant in the dioecious persimmon *Diospyros lotus* [38]. Furthermore, many other TFs including WRKY, F-box, SPL, GATA, YABBY, and DELLA have been implicated in various processes of plant development and floral differentiation [19,20].

## Characterization of Sex-Linked Genes through Next-Generation Sequencing

The complex and dynamic event of floral development depends on the tight regulation of gene expression and controlled environmental cues [39]. In recent years, several studies have been reported regarding floral development and whorl speciation in hermaphrodites, as well as unisexual plants of model and non-model species [40-45]. However, the majority of these studies are based on mutant analysis. For example, a sex determination gene (*TASSELSEED2*) in maize encodes a short-chain alcohol dehydrogenase required for stage-specific floral organ abortion [46], and a conserved mutation in the active site of 1-aminocyclopropane-1-carboxylic acid synthase leads to andromonoecy in melons [47]. However, sex differentiation is a complex phenomenon in angiosperms, with the involvement of several genes that are differentially expressed in diverse tissues and developmental phases [48]. Under these circumstances, the identification and characterization of a few sex-linked genes at a particular stage may not be able to provide the entire mechanism of sex determination in a given species [49]. In other words, it is essential to characterize myriads of genes from different developmental stages of dioecious species to understand the complexity of plant sex determination. Large-scale gene expression analysis methods such as mRNA differential display, suppression subtractive hybridization, reverse-transcription polymerase chain reaction, and microarrays have been previously used to assess the vital stages of sex determination in a wide range of plants [50,51]. However, these methods had limited applications for understanding sex determination due to their poor sensitivity, the inconvenience of cross-hybridization, and the non-availability of the total genome sequences. The advent of next-generation RNA-sequencing (RNA-Seq) technology has offered a powerful, economical, and highly sensitive method for the discovery of novel transcripts and the assessment of transcriptome dynamics [52]. Moreover, a *de novo* assembly of RNA-Seq reads can be efficiently used for gene discovery in non-model plant species where the total genome information is unavailable [53].

Next-generation sequencing (NGS) technologies have facilitated gene discovery and the global analysis of molecular mechanisms related to growth and development in numerous plant species, including members of the Cucurbitaceae family. Transcriptome profiling and comparison between gynoecious and hermaphrodite cucumber plants resulted in the identification of 200 differentially expressed genes (DEGs) with a significant role in plant sex determination process [54]. In another study, Solexa sequencing was performed to determine the transcript profile of apical tissues from a gynoecious mutant and a monoecious wild type of cucumber [40]. A total of 143 upregulated and 600 downregulated genes were identified in the mutant type. The study suggested that multiple genes from plant hormone signaling pathways, including *ACS, Asr1, CsIAA2, CS-AUX1, TLP*, and *EREBP*, play critical roles in sex determination and floral development in cucumbers. Similarly, RNA-Seq analysis of two near-isogenic lines of melons (male sterile line DAH3615-MS and male fertile line DAH3615) resulted in the identification of 1,259 DEGs significantly associated with male fertility [55]. The majority of these genes were linked to pathways related to pollen development, stamen development, and pollen tube elongation.

Among other plant species, a genome-wide high-throughput transcriptomic sequencing for young floral buds of sterile and fertile plants of *Brassica napus* and subsequent mapping onto the AA and BB genomes revealed a total of 3231 genes of *B. rapa* and 3,371 genes of *B. oleracea* with considerable differential expression levels [56]. That study reported 760 DEGs specific to fertile and 44 DEGs specific to sterile plants. After Gene Ontology (GO) annotation, 11 DEGs were identified as involved in pollen wall assembly (GO: 0010208), of which three DEGs were beta-1,3-glucanase genes (Bra028343, Bra037057, Bra038969) implicated in male gametophyte development and pollination. Similarly, 454 pyrosequencing and a comparative analysis during the development of male and female flowers of the monoecious species *Quercus suber* revealed DEGs in the early and late stages of development of female and male flowers, some of which were shown to be involved in pollen development, ovule formation, and flower development of other species with a monoecious, dioecious, or hermaphroditic sexual system [57]. Interestingly, a homolog for *POLYGALACTURONASE-1*, which is expressed 356 times more in female tissues, has been previously associated with pollen [58-60] and carpel development [61]. Another gene, *QsENDO-BETA-1,3-1,4 GLUCANASE*, a member of the glycoside hydrolase family, which is 199 times more expressed in female samples, has been linked to male sterility due to defects in anther dehiscence [62]. Illumina sequencing of inflorescent meristems and the flowering stages of sugar apples (*Annona squamosa L.*) resulted in 71,948 unigenes, 147 of which were represented by various TF families involved in floral transition and development [63]. Likewise, different NGS platforms and diverse sequencing chemistries have been utilized to characterize DEGs in the male and female *Salix suchowensis* [64], DEGs linked with pistil abortion in Japanese apricots [65], genes linked to sex type differentiation in *Ginkgo biloba L.* [66] and genes associated with the regulatory mechanism of floral development in olive (*Oleaeuropaea L.*) [67]. Most recently, RNA-Seq analysis was performed to study the floral bud differentiation in *Magnolia sinostellata* [68]. The study revealed 82 genes out of a total of 11,592 DEGs involved in flowering and 20 genes were found to be critically involved in bud differentiation at different stages of flower development. Overall, these studies suggest that NGS analysis and the associated bioinformatics components have laid the foundation for the genome-wide characterization and functional prediction of genes linked to floral development and sex differentiation in angiosperms.

## Conclusion

To understand the complex and dynamic event of floral development, research in floral evolution and development is using a combination of approaches to elucidate the genetic basis for the enormous diversity in floral morphology. The need of the hour is to understand how this variation has contributed to the radiation of angiosperms. Sex-linked genes, especially TFs, are key players in flower development, and further research needs to be done in this promising area to comprehend the event of floral development.

## Figures and Tables

**Table 1.** Major sex-linked genes characterized in different plant species

Gene	Symbol	Species	Function
*AGAMOUS-LIKE 65*,	*AGL65, 66,104*	*Arabidopsis thaliana*	Pollen maturation and tube growth
*66*, *104*			
*AGAMOUS*	*AG*	*Arabidopsis thaliana*	Homeotic C-class gene; carpel and stamen specification, lineage-specific sub functionalization of the homeotic C function; fruit development (e.g., tomato versus *Arabidopsis*)
*SHATTERPROOF 1*, *2 *	*SHP1*, *2*	*Arabidopsis thaliana*	Carpel, ovule, and fruit development; dehiscence; periodic lateral root formation
*APATELA 3, PISTILATA *	*AP3, PI*	*Arabidopsis thaliana*	Floral homeotic B function, specification of petaloid organs
FLORAL LOCUS C	*FLC*	*Arabidopsis thaliana*	Potential role in floral bud dormancy; perennial life history in *Arabis alpina*
SQUAMOUS	*SQUA*	*Antirrhinum majus*	Floral meristem and organ identity specification; floral transition; fruit development
			
	*STMADS11*	*Arabidopsis thaliana*	Control of floral transition; repression of precocious homeotic gene expression
*AGAMOUS-LIKE 2*	*AGL2*	*Arabidopsis thaliana*	Floral homeotic E function
*A BSISTER *	*ABS*	*Arabidopsis thaliana*	Endothelium development in seeds
*SEEDSTICK *	*STK*	*Arabidopsis thaliana*	Carpel and ovule development; periodic lateral root formation
*XAANTAL1 *	*XAL1*	*Arabidopsis thaliana*	Transition to flowering
*MADS AFFECTING*	*MAF5*	*Arabidopsis thaliana*	Transition to flowering (activator)
*FLOWERING 5*			
*CAULIFLOWER *	*CAL*	*Arabidopsis thaliana*	Meristem identity specification
*FRUITFULL *	*FUL*	*Arabidopsis thaliana*	Meristem identity specification; annual life cycle regulator, with SOC1; fruit development; cauline leaf growth
*AGAMOUS-LIKE 24 *	*AGL24*	*Arabidopsis thaliana*	Transition to flowering (activator)
*SHORT VEGETATIVEPHASE*	*SVP*	*Arabidopsis thaliana*	Transition to flowering (repressor)
*APETALA1 *	*AP*	*Arabidopsis thaliana*	Meristem identity specification; homeotic A-class gene
*DIANA *(*AGAMOUS-LIKE 61*)	*DIA*	*Arabidopsis thaliana*	Central cell and endosperm development
*AGAMOUS-LIKE 23*	*AGL23*	*Arabidopsis thaliana*	Embryo sac development
*SUPPRESSOR OF*	*SOC1*	*Arabidopsis thaliana*	Transition to flowering (activator); periodic lateral root formation
*OVEREXPRESSION OF CONSTANS 1*			
*AP3 MADS box gene*	*SLAP3*	*Silene latifolia*	Male floral bud development
*DUF538 *	*MROS3X*	*Silene latifolia*	Male floral bud development
*PLENA*	*PLE*	*Antirrhinum majus*	Specify stamen and carpel identity
*Floral binding protein gene*	*FBP7, FBP11*	*Petunia hybrida*	Ovule identity
			
*FARINELLI*	*FAR*	*Antirrhinum majus*	Male fertility
*PrMADS1, PrMADS1, PrMADS2*	*PRMADS1,2,3*	*Pinus radiata *	Petal, stamen, and carpel development, and preventing the indeterminate growth of the flower meristem.
			
*DEFICIENS/GLOBASA*	*DEF/GLO*	* Antirrhinum majus*	Petal and stamen identity
*Gerbera MADS box gene*	*GRCD1*	*Gerbera hybrida*	Stamen development and identity
			
*Zea Agamous 3*	*ZAG3*	*Zea mays*	Carpel development
*ZmMADS1*	*ZMM5*	*Zea mays*	Expressed during flower development: in egg cells and embryos
*CmWIP1*	*CmWIP1*	*Cucumis melo*	Stamen development in male flowers and suppression of carpel development
*CmACS7*	*CmACS7*	*Cucumis melo*	Stamen suppression in female flower development
*OGI*	*OGI*	*Diospyros lotus*	Suppression of anther development in female flowers
*Domain of unknown function 247*	*DUF 247*	*Asparagus officinalis*	Suppressor of pistil development
*RADIALIS*	*RAD1 and RAD2*	*Rumex acetosa*	Stamen whorl development
*Ras-proximate-1 or Ras-related protein 1*	*RAP1*	*Rumex acetosa*	Carpel and stamen whorl specification/ development
*TASSELSEED2*	*ts2*	*Zea mays*	Male sex determination and stamen development
*STABILIZED1*	STA1	*Silene latifolia*	Tapetum development in male flowers
*Silene latifolia MADS1*	*SLM1*	*Silene latifolia*	Specify stamen and carpel identity
*Silene latifolia MADS2*	*SLM 2*	*Silene latifolia*	Developing stamens of smut-infected
			female flowers
*Silene latifolia MADS3*	*SLM 3*	*Silene latifolia*	Repression of gynoecium development in male flowers
*AGAMOUS-LIKE 80*	*AGL80*	*Arabidopsis thaliana*	Central cell and endosperm development
*Silene latifolia MADS4,5*	*SLM 4,5*	*Silene latifolia*	Floral meristem and organ identity specification; floral transition; fruit development
*Silene latifolia sepallata 1 and 3*	*SlSEP1 and SlSEP3*	*Silene latifolia*	Expressed in young flower meristems, developing petals, male anthers, and female ovules
